# Supplementary material for: Etv6 activates vegfa expression through positive and negative transcriptional regulatory networks in Xenopus embryos
Source: Nat Commun. 2019 Mar 6;10:1083. doi: 10.1038/s41467-019-09050-y (PMC6403364; doi:10.1038/s41467-019-09050-y)
Supplement: Supplementary file 1 — Supplementary Information [file 41467_2019_9050_MOESM1_ESM.pdf]

Supplementary Information

Etv6 activates *vegfa* expression through positive and negative transcriptional regulatory networks in *Xenopus* embryos

Li *et al*.

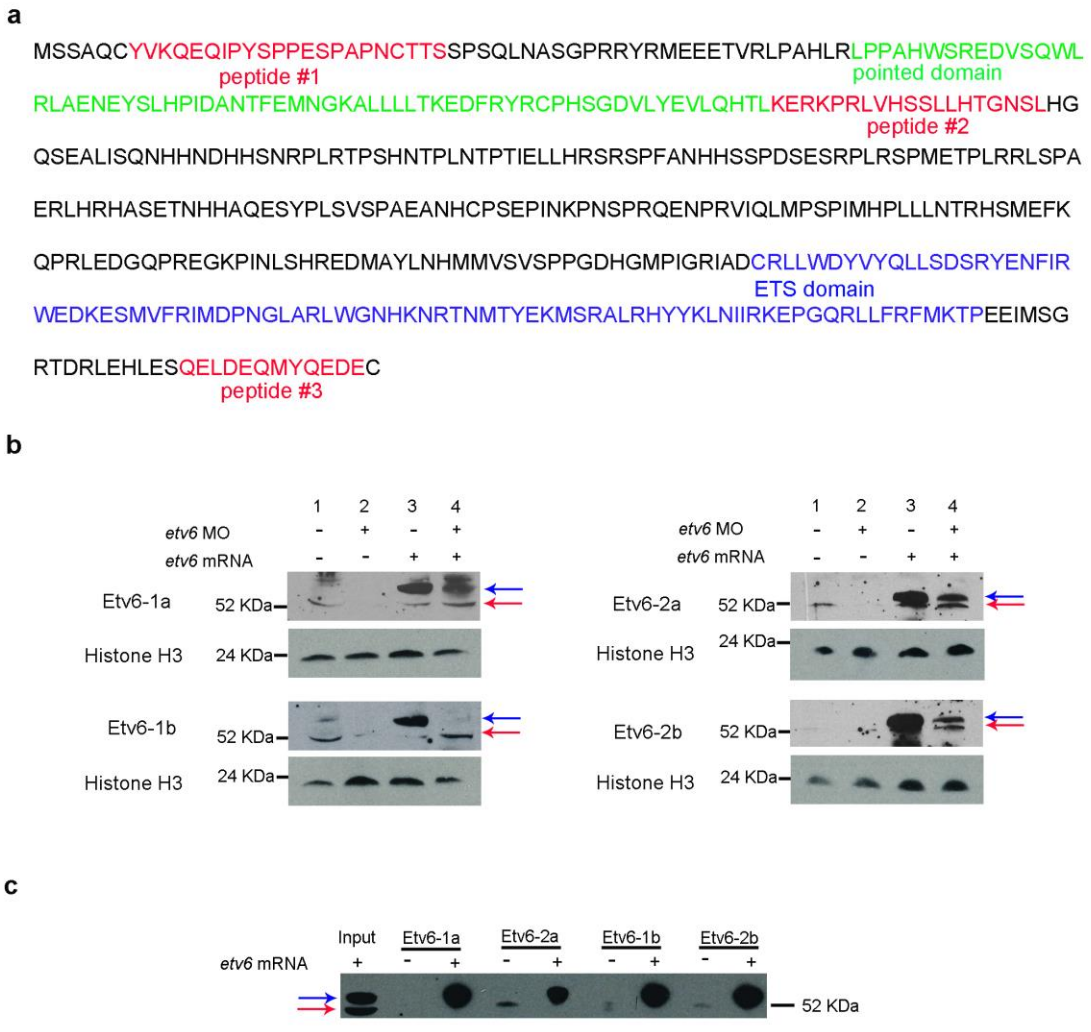

Supplementary Figure 1. Generation and characterization of ChIP grade antibodies against *Xenopus* Etv6.

(**a**) Amino acid sequence for *Xenopus* Etv6 (Accession Number NP_001124423.1). Pointed domain and ETS domain are indicated in green and blue, respectively. The sequences of the three peptides used for the generation of antibodies are indicated in red. Two rabbit polyclonal antibodies were obtained per peptide: Etv6-1a, -1b, -2a -2b, -3a, -3b.

(**b**) Western blot validating the specificity of Etv6 antibodies. Protein extracts from stage 22 wild-type (WT), Etv6-deficient (*etv6* MO), *etv6*-overexpressing (*HA-etv6* mRNA) or Etv6-deficient + *etv6*-overexpressing (*etv6* MO+*HA-etv6* mRNA) somites were used for western blot analysis. While polyclonal antibodies generated from

peptide 1 (Etv6-1a and Etv6-1b) and peptide 2 (Etv6-2a and Etv6-2b) detect both endogenous and exogenous expression of Etv6, polyclonal antibodies generated from peptide 3 (Etv6-3a and Etv6-3b) did not detect expression of Etv6 and cross-reacted with an unknown protein (data not shown). Lane 1, WT; lane 2, 40 ng *etv6* MO; lane 3, 1.0 ng *etv6* mRNA; lane 4, 40 ng *etv6* MO + 1.0 ng *etv6* mRNA. The predicted molecular weight of Etv6 is around 52 KDa. (http://web.expasy.org/compute_pi/). Blue and red arrows point to exogenous and endogenous expression of Etv6, respectively.

(**c**) Immunoprecipitation assay validating the affinity of anti *Xenopus* Etv6 antibodies to endogenous Etv6. Protein extracts from the somites of stage 22 WT embryos were used for immunoprecipitation; somite extracts from embryos injected with exogenous *etv6* mRNA were used as positive control. Polyclonal antibodies generated from peptide 1 (Etv6-1a and Etv6-1b) and peptide 2 (Etv6-2a and Etv6-2b) were tested for their capacity to immunoprecipitate Etv6 protein. Western blot was performed using antibody Etv6-1b as primary antibody. Blots show that antibodies Etv6-2a and Etv6-2b could immunoprecipitate endogenous Etv6 protein. Antibody Etv6-2a showed greater affinity to Etv6 and was selected for subsequent experiments. Blue and red arrows point to exogenous and endogenous expression of Etv6, respectively.

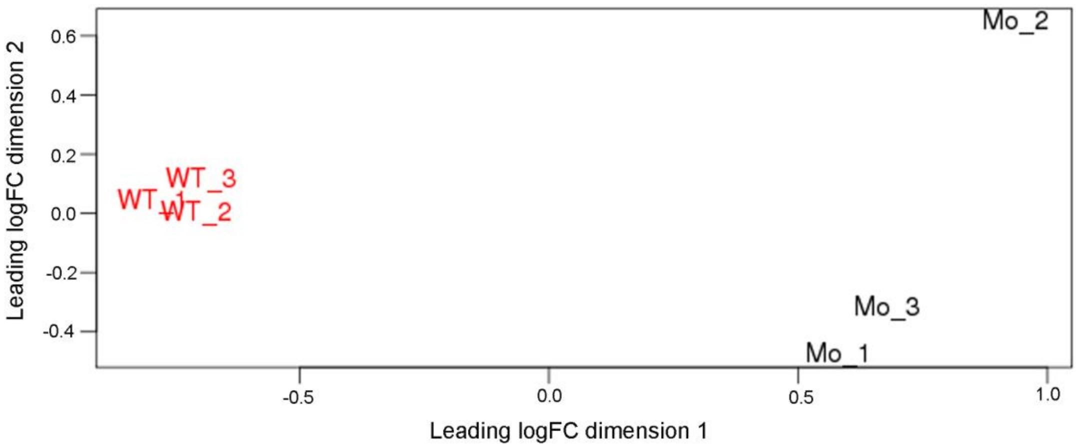

Supplementary Figure 2. PCA plot illustrating the significant differences in the transcriptome of Etv6-deficient (*etv6* MO) somites when compared to that of wild type (WT) somites.

Three independent biological replicates were used for each condition.

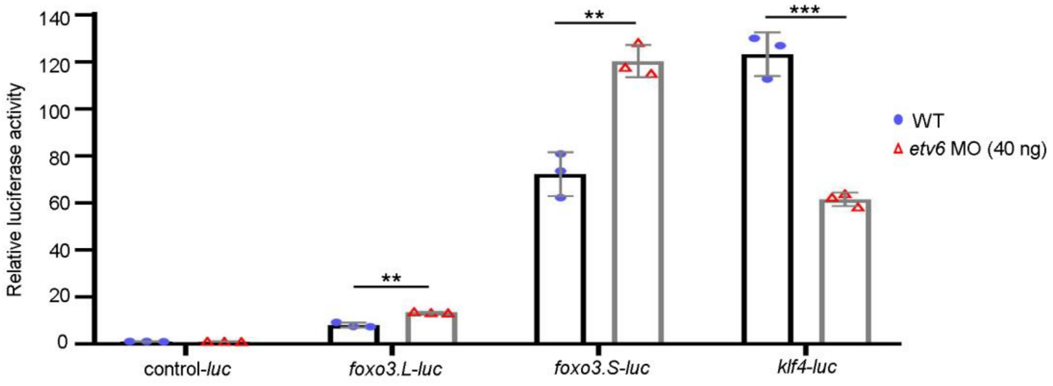

Supplementary Figure 3. The sequences underlying the Etv6 peaks in the promoters of *klf4* and *foxo3* exhibit positive and negative transcriptional activities, respectively.

Luciferase reporter assays were used to test the transcriptional activity of Etv6-bound sequences in *klf4* and *foxo3* promoters. The vectors (control-*luc, foxo3.L-luc*, *foxo3.S-luc* and *klf4-luc*) were injected in 2-cell stage embryos on their own or co-injected with *etv6* MO to perform analyses on wild-type (WT) or Etv6-deficient backgrounds, respectively. Luciferase activity was measured on stage 22 somitic material. In comparison to empty luciferase vector (control-*luc*), the three Etv6-bound sequences induced the expression of the luciferase reporter in WT embryos. The transcriptional activities of the *foxo3* promoter Etv6 peak were significantly upregulated in Etv6-deficient embryos. In contrast, the activity of the *klf4* promoter Etv6 peaks was significantly downregulated. This indicates that Etv6 activates *klf4* transcription while repressing *foxo3*. Error bars represent SEM of three biological replicates. P(*foxo3.L*)=0.002, P(*foxo3.S*)=0.007, P(*klf4*)=0.0007, two-tailed Student’s t-test.

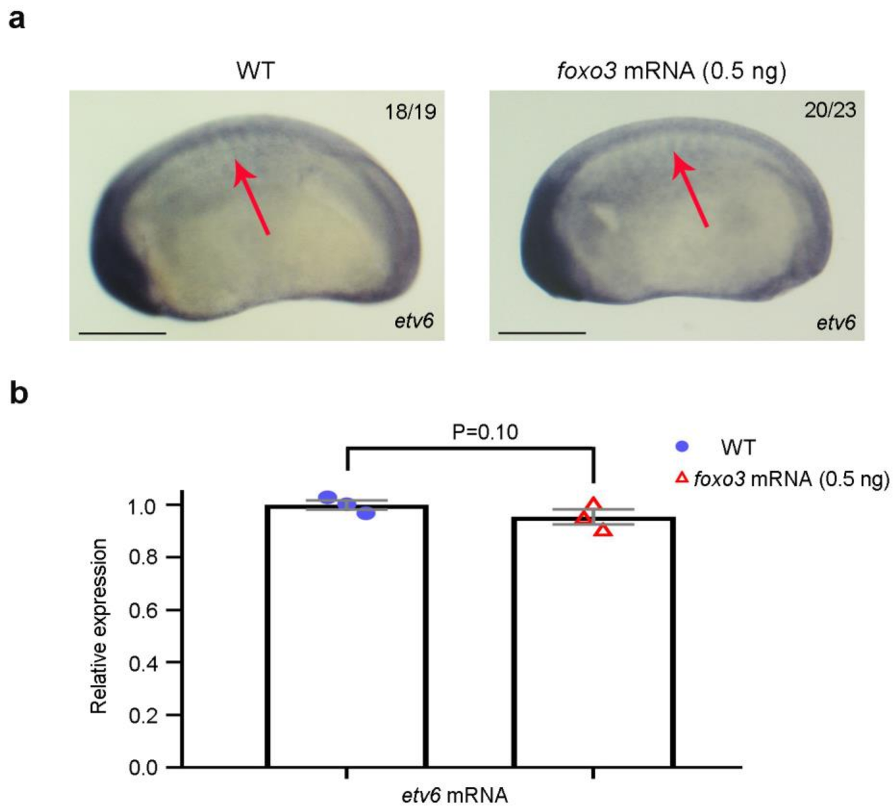

Supplementary Figure 4. *Etv6* expression in the somites is not affected in *foxo3* overexpressed embryos.

(**a**) WISH showing *etv6* expression in the somites (arrows) of stage 22 WT, and *foxo3* overexpressed embryos. Embryos are shown in lateral view with anterior to the left and dorsal to the top. Numbers in top right corner indicate the number of embryos exhibiting the phenotype pictured (scale bars: 0.5 mm).

(**b**) RT-qPCR confirming that similar levels of *etv6* mRNA are expressed in the somites of WT and *foxo3* overexpressed embryos at stage 22. Expression was normalized to *odc1*. Error bars represent SEM of three biological replicates.

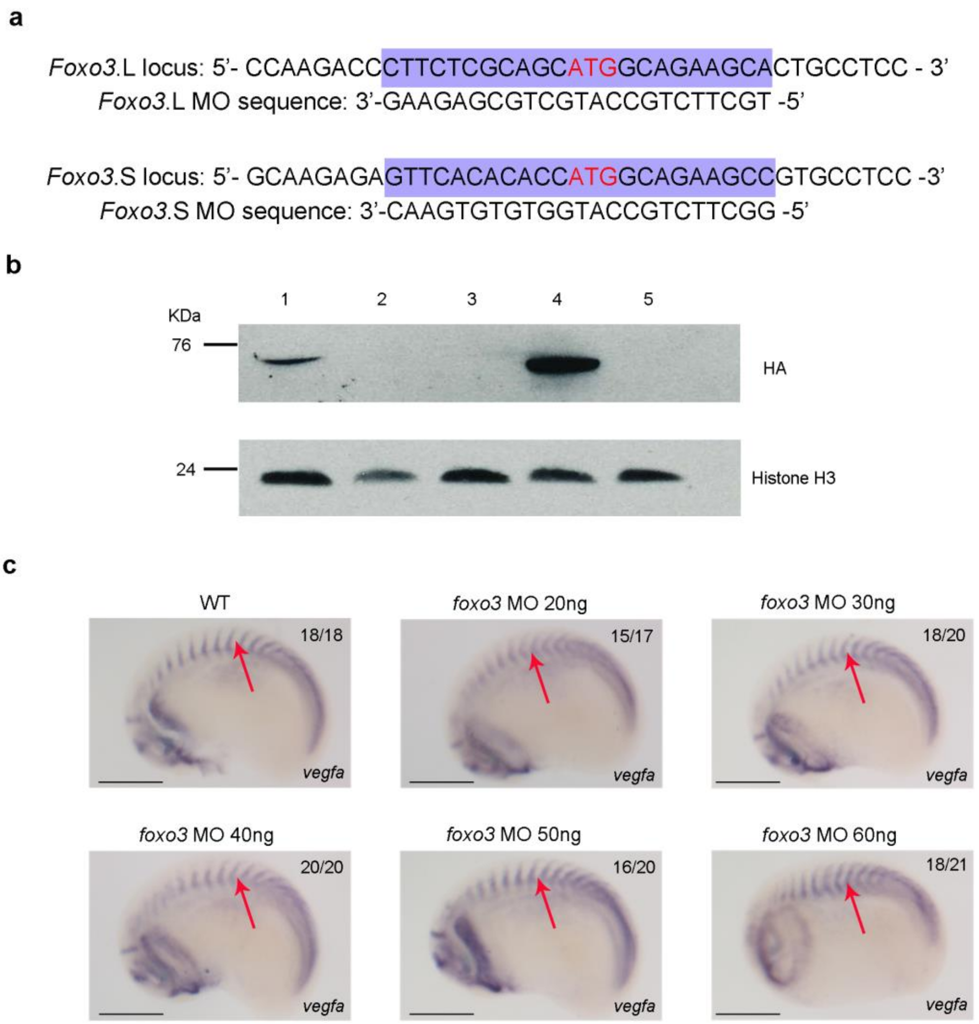

Supplementary Figure 5. *Foxo3* deficiency has no effect on *vegfa* expression in the somites at stage 22 of development.

(**a**) cDNA alignment showing the sequence differences between *foxo3.L* and *foxo3.S*. Due to lack of homology in the region of the transcription starting codon, a single MO targeting both genes could not be generated. Therefore, two MOs, each one targeting a specific *foxo3* gene, were designed; their target sequence is highlighted in purple. (**b**) Western blot showing that *foxo3* MOs block efficiently the translation of their targets. To assess the efficiency of the *foxo3* MOs, the cDNAs for *foxo3.L* and *foxo3.S*, containing the sequences targeted by the MOs, were HA-tagged, mRNA was synthesised *in vitro* and injected into 2-cell stage embryos (0.5 ng per embryo). Translation of these cDNAs was blocked when co-injected with their corresponding

blocking MO (30 ng). Lane 1, 0.5 ng *foxo3.L-HA*; lane 2, 0.5 ng *foxo3.L-HA* + 30 ng *foxo3.L* MO; lane 3, uninjected; lane 4, 0.5 ng *foxo3.S-HA*; lane 5, 0.5 ng *foxo3.S-HA* + 30 ng *foxo3.S* MO. Histone H3 was used as a loading control.

(**c**) WISH showing that *vegfa* expression in the somites (arrows) is not affected in *foxo3*-deficient embryos. *Foxo3* depletion was performed by co-injecting *foxo3.L* MO and *foxo3.S* MO in a 1:1 ratio to the total concentrations indicated. Images show stage 22 embryos in lateral view with anterior to the left and dorsal to the top. Numbers in top right corner indicate the number of embryos exhibiting the phenotype pictured (scale bars: 0.5 mm).

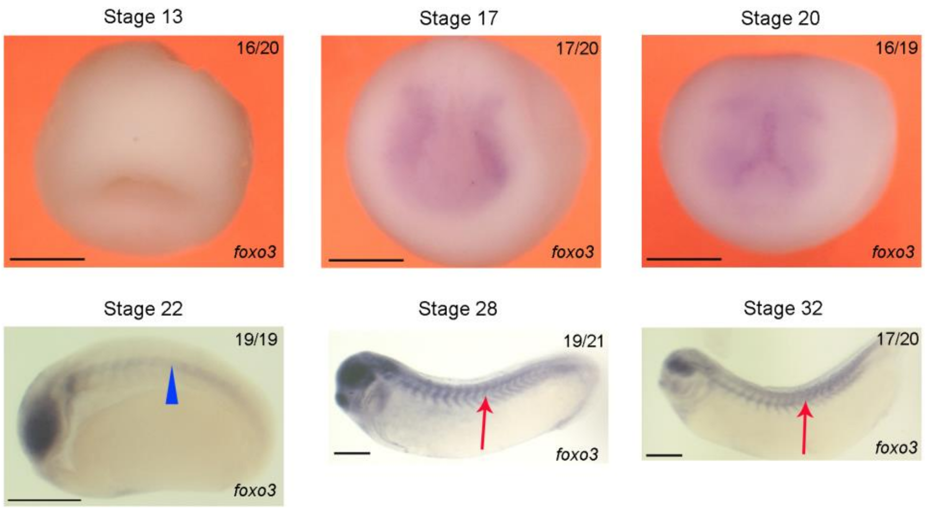

Supplementary Figure 6. WISH showing the expression pattern of *foxo3* during early *Xenopus* development.

Foxo3 mRNA is not detected before stage 22 of development. At stage 22, its expression is detected in the notochord (arrowhead). Expression in the somites is detected at stage 28, i.e. after the establishment of definitive hemangioblast in the lateral plate mesoderm. Arrows in stage 28 and 32 embryos indicate expression in the somites. Stage 13-20 embryos are shown in anterior view with dorsal to the top. Stage 22-32 embryos are shown in lateral view with anterior to the left and dorsal to the top. Numbers in top right corner indicate the number of embryos exhibiting the phenotype pictured (scale bars: 0.5 mm).

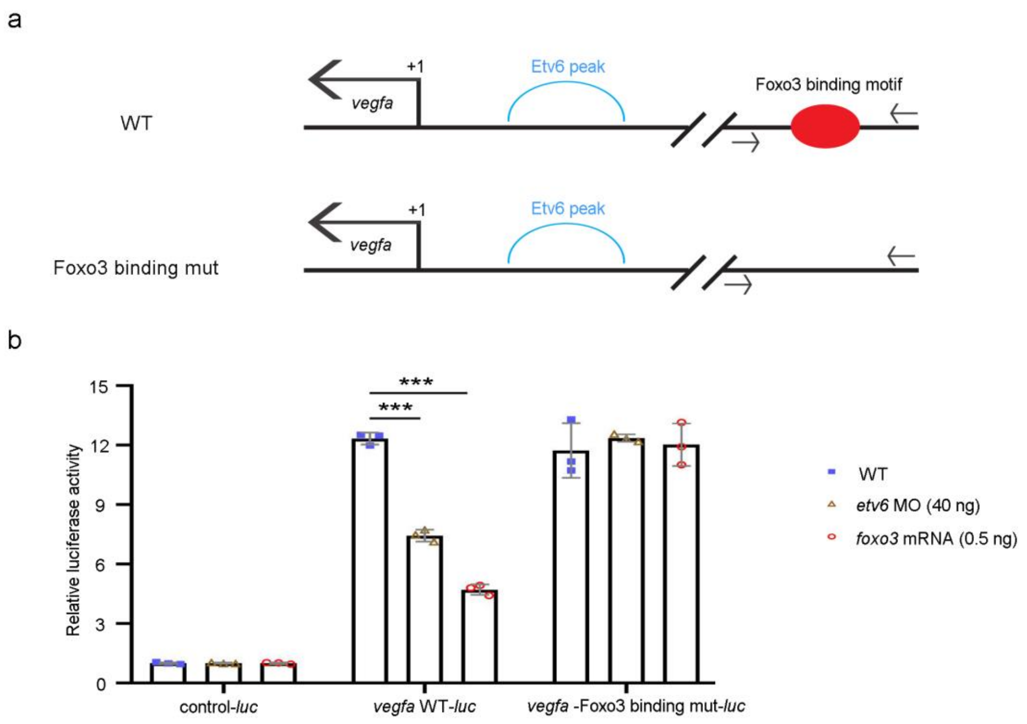

Supplementary Figure 7. The Foxo3 binding motif controls the transcriptional activity of the *vegfa* promoter.

(**a**) Schematic diagram depicting the location of the conserved Foxo3 binding site in the *Xenopus vegfa* promoter. The location of the Etv6 ChIP peak is indicated in blue. Bottom diagram illustrates the Foxo3 binding site-deleted form in the *vegfa* promoter. Arrows indicate the sequences tested in luciferase assays.

(**b**) Luciferase reporter assays were used to test the transcriptional activity of sequences containing the wild type Foxo3 binding motif (5'-GTAAACA-3') (*vegfa* WT-*luc*) or deleted of Foxo3 binding motif (*vegfa*-Foxo3 binding mut-*luc*), as depicted in (a). The vectors were injected in 2-cell stage embryos on their own or co-injected with *etv6* MO or *foxo3* mRNA to perform analyses on wild-type (WT), Etv6-deficient or *foxo3* overexpressing backgrounds, respectively. Luciferase activity was measured on stage 22 somitic material. Note that luciferase activity driven by wild-type *vegfa* promoter sequences is significantly reduced in Etv6-deficient embryos or when *foxo3* is overexpressed when compared to WT embryos. In contrast, when the Foxo3 binding motif is deleted from this sequence, luciferase activity is no longer repressed in Etv6-deficient embryos or when *foxo3* is overexpressed. Error bars represent SEM

of three biological replicates. ***P(WT/*etv6* MO)= 3.0969E-05, ***P(WT/*foxo3* mRNA)= 4.44051E-06, two-tailed Student’s t-test.

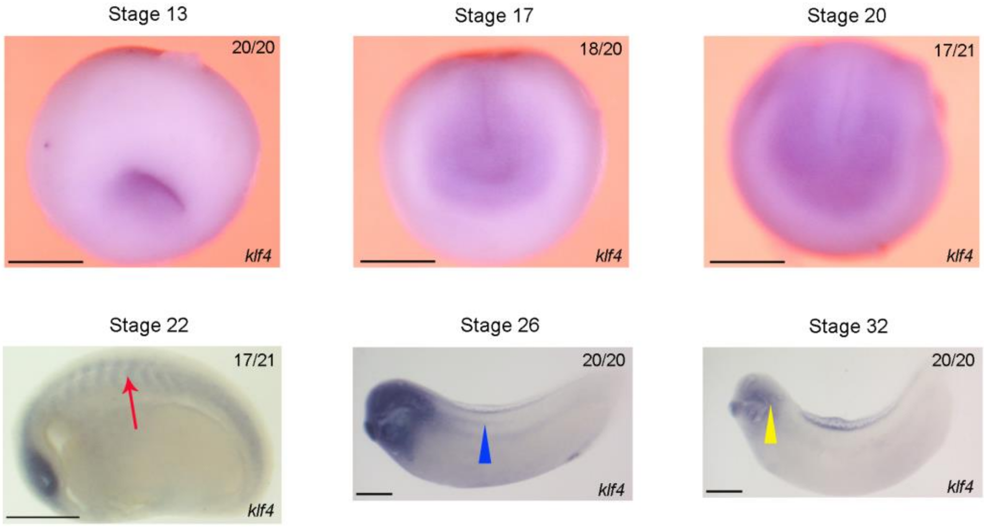

Supplementary Figure 8. WISH showing that *klf4* is transiently expressed in the somites of *Xenopus* embryos.

Klf4 mRNA is not detected before stage 22 of development. At stage 22, its expression is detected in the somites (red arrow) but this expression is extinguished by stage 26 (blue arrowhead indicates expression in the notochord). Yellow arrowhead in stage 32 embryo indicates expression in the head. Stage 13-20 embryos are shown in anterior view with dorsal to the top. Stage 22-32 embryos are shown in lateral view with anterior to the left and dorsal to the top. Numbers in top right corner indicate the number of embryos exhibiting the phenotype pictured (scale bars: 0.5 mm).

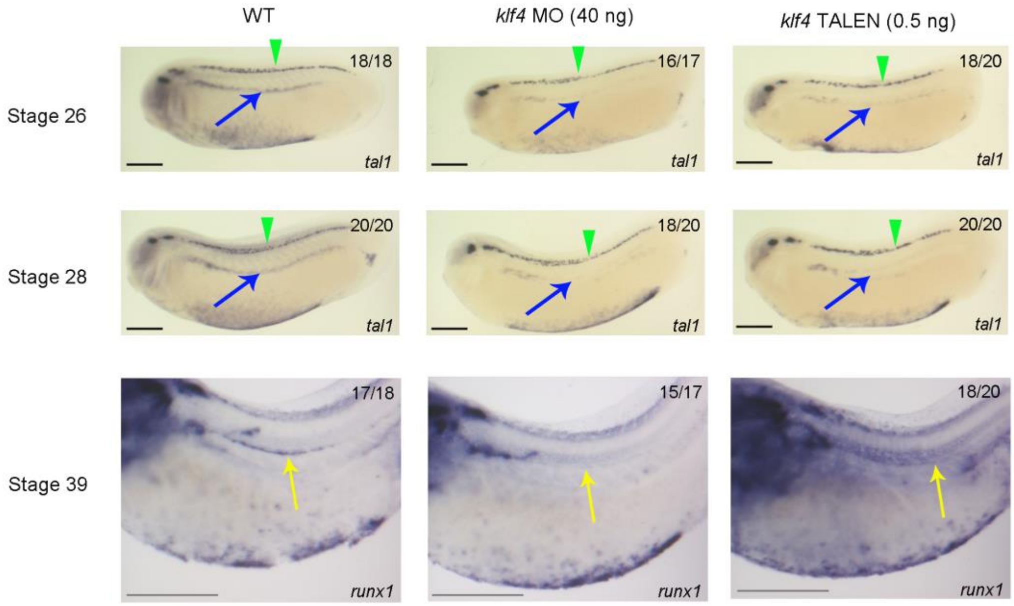

Supplementary Figure 9. *Klf4* is required for the emergence of HSC.

WISH demonstrating that the establishment of definitive hemangioblasts in the lateral plate mesoderm, as indicated by *tal1* expression (arrows in stage 26 and 28 embryos), and hemogenic endothelium in the ventral wall of the dorsal aorta, as indicated by *runx1* expression (arrows in stage 39 embryos), is impaired in *klf4*-deficient embryos. Arrowheads in stage 26 and 28 embryos indicate expression of *tal1* in neurons, which is unaffected by *klf4* depletion. Embryos are shown in lateral view with anterior to the left and dorsal to the top. Numbers in top right corner indicate the number of embryos exhibiting the phenotype pictured (scale bars: 0.5 mm).

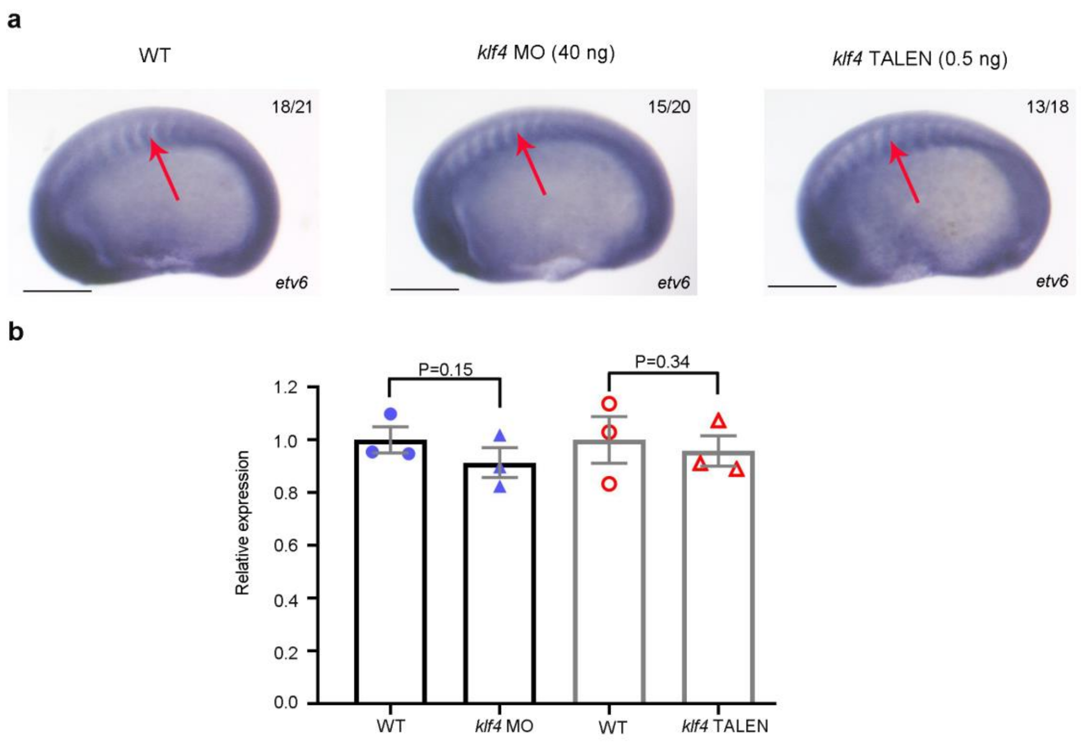

Supplementary Figure 10. *Etv6* expression in the somites is not affected in *klf4*-deficient embryos.

(**a**) WISH showing *etv6* expression in the somites (arrows) of stage 22 WT, *klf4* MO- and *klf4* TALEN-injected embryos. Embryos are shown in lateral view with anterior to the left and dorsal to the top. Numbers in top right corner indicate the number of embryos exhibiting the phenotype pictured (scale bars: 0.5 mm).

(**b**) RT-qPCR confirming that similar levels of *etv6* mRNA are expressed in the somites of WT and *klf4*-deficient embryos at stage 22. Expression was normalized to *odc1*. Error bars represent SEM of three biological replicates.

a

ATTATCCAAAAGTTACACGCACACCGAAGCAATTCTCTTTTAAGATTTATTAGAAAAATAATTAA
TGAAACATCACATCGACGATTCGATTCCTGGTTTAAGCCTTTCTCAAGATGCTTCATCTCTATA
GAGAAGGCAGGCTGCAGCCTCAGTGTCACATAACTAGGGGCGGCGGCGAGGGAGTGGCTTC
AGTGAGGGCGGGGCTTTGCTCAATTGGCTAGCCTGTGAGCAGAACCGGAGCTGACACAGAC
AGAAGAACGCAGTGTAAGAAGAGAGACCCGGGAACAGGGAAGGGCTGAATTGCCGTTGTATA
GGACGGTACAAGGCTACAGGGCACAGCAGGATTATTCACTCACGGTTGGCATATAGAGCAGG
CACTGAGTGCTGGAGATCGGGACCGGGGCATTTACGCGCGATGCATTGAGAGTTGGCGGAA
CTTTCCTGGGATCTTGTTACATTGTATCCGTGTCTGTTACGTTGTATCAACCAGTGGAGCCGTA
GGTTTTACCCAAAAGGATTATGTGGCCGCTGCTTCCTGCTCTGGGGGCTGAGTGAGGGCCCG
TCCAATCGTTGCTCATTTACCTACTGGGGGAGCCGGGACTTCTCCCTAATCTTGGGTTCCCCC
CACTCCCTGAGCCCCTCACCGGAAAATCATG

TSS

b

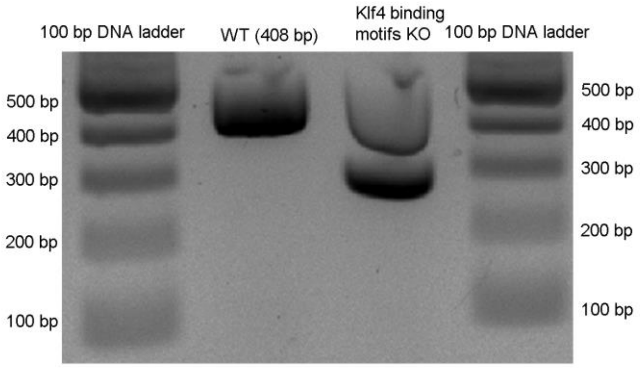

c

mutation #1: GAGCAGAACCG 11 bp deletion

mutation #2: TGAGCAGAACC 11 bp deletion

mutation #3: AGCCTGTGAGCAGAACCGG 19 bp deletion

mutation #4: AAGCCTTTCTCAAGATGCTTCATCTCTATAGAGAAGGCAGGCTGCAGCCTCAGT
GTCACATAACTAGGGGCGGCGGCGAGGGAGTGGCTTCAGTGAGGGCGGGGC
TTTGCTCAATTGGCTAGCCTGTGAGCAGAAC 136 bp deletion

Supplementary Figure 11. Klf4 binding motifs in the *vegfa* promoter region were deleted using TALENs.

(**a**) DNA sequence for the *X. laevis vegfa* promoter (-653 bp ~ +3 bp) containing the Etv6 ChIP peak. The Etv6-peak sequence is indicated in red; Klf4 binding motifs under Etv6-peak are indicated in blue; the transcription start site is indicated in green; the TALENs designed for deletion of the Klf4 binding motifs are indicated in yellow and purple, respectively.

(**b**) TALEN activity generated a range of deletions in the *vegfa* promoter. Genomic DNA was obtained from 20 WT and TALEN-injected stage 22 somites and PCR-amplified. The smaller product detected from TALEN-injected somites when

compared to WT somites (~270 bp versus ~400 bp) represents the majority of the mutated sequences, as shown in (**c**).

(**c**) Sequences showing the range of mutations caused by TALEN injection. The PCR products obtained in (**b**) were cloned and Sanger sequenced. TALENs caused mutations in 100% of the clones sequenced (10/10). Mutation #1 was detected in two clones, mutation #2 in two clones, mutation #3 in one clone, and mutation #4 in five clones (50%).

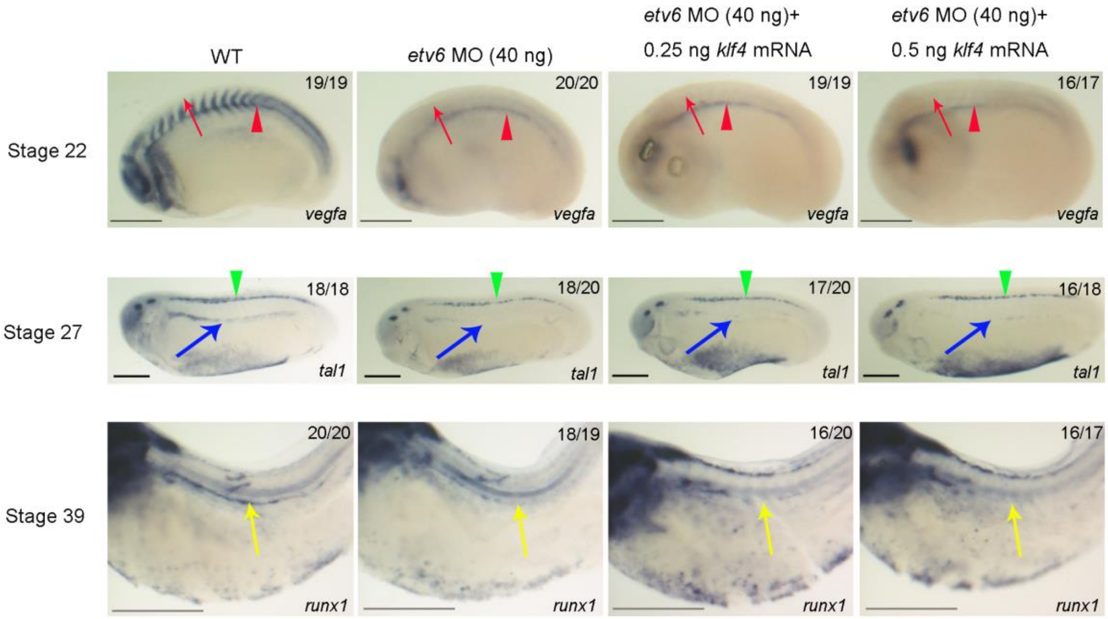

Supplementary Figure 12. *Klf4* overexpression does not rescue the hematopoietic phenotype of Etv6-deficient embryos.

WISH showing that *vegfa* expression in *Xenopus* somites (arrows in stage 22 embryos), *tal1* expression in definitive hemangioblasts in the lateral plate mesoderm, (arrows in stage 27 embryos), and *runx1* expression in hemogenic endothelium in the ventral wall of the dorsal aorta (arrows in stage 39 embryos) in wild-type (WT) embryos, Etv6-deficient (*etv6* MO) embryos and in embryos deficient for Etv6 and overexpression *klf4* mRNA (*etv6* MO+*klf4* mRNA). Note that *klf4* overexpression does not rescue the hematopoietic phenotype of Etv6-deficient embryos. Arrowheads in stage 22 embryos indicate expression of *vegfa* in the hypochord, which is unaffected by Etv6 depletion. Arrowheads in stage 27 embryos indicate expression of *tal1* in neurons, which is unaffected by Etv6 depletion. Embryos are shown in lateral view with anterior to the left and dorsal to the top. Numbers in top right corner indicate the number of embryos exhibiting the phenotype pictured (scale bars: 0.5 mm).

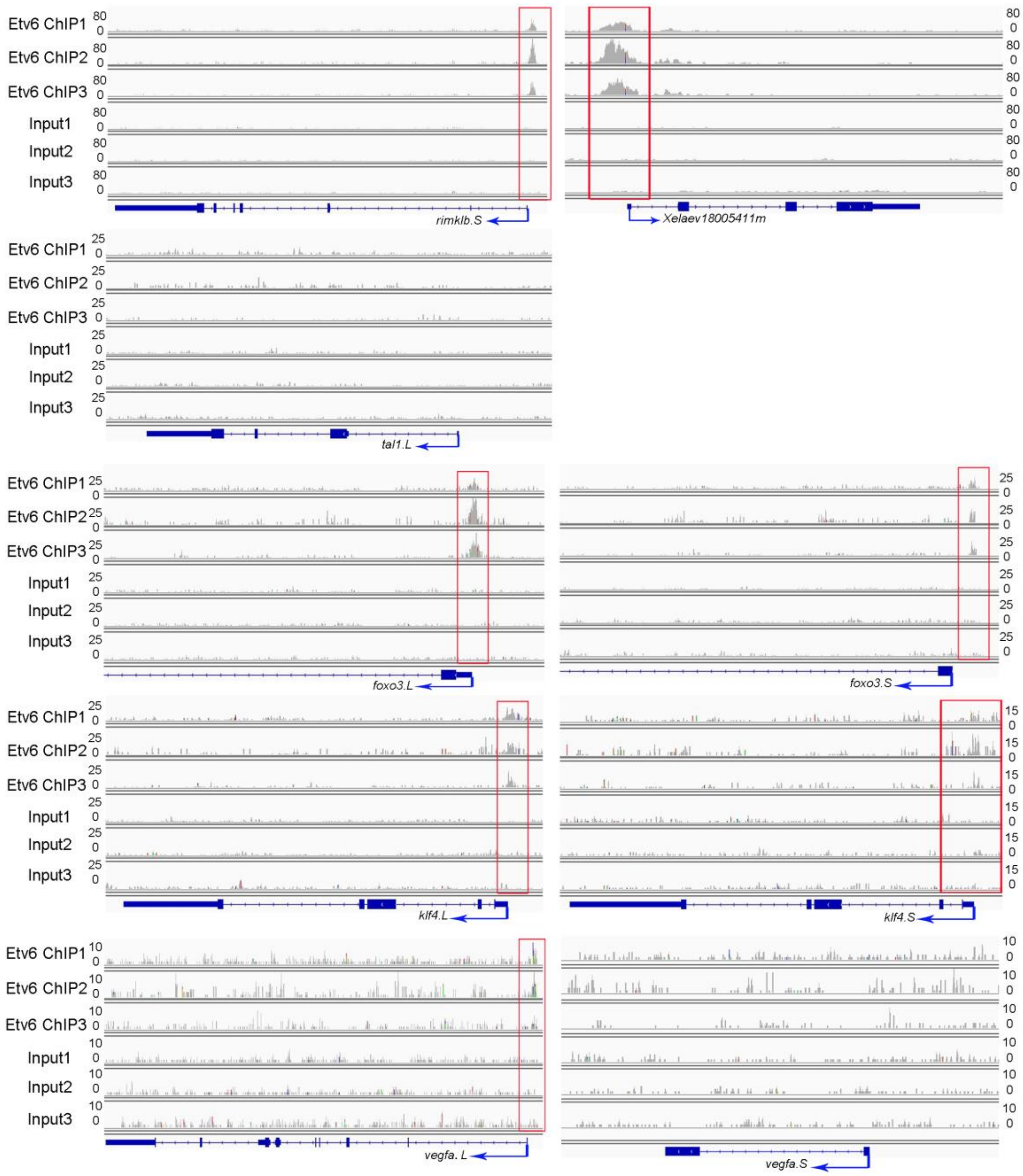

Supplementary Figure 13. Etv6 ChIP-seq peaks and input tracks from individual ChIP-seq replicates 1, 2 and 3 corresponding to the genomic loci discussed in this study: *rimklb*, *Xelaev18005411m*, *tal1*, *foxo3*, *klf4* and *vegfa*.

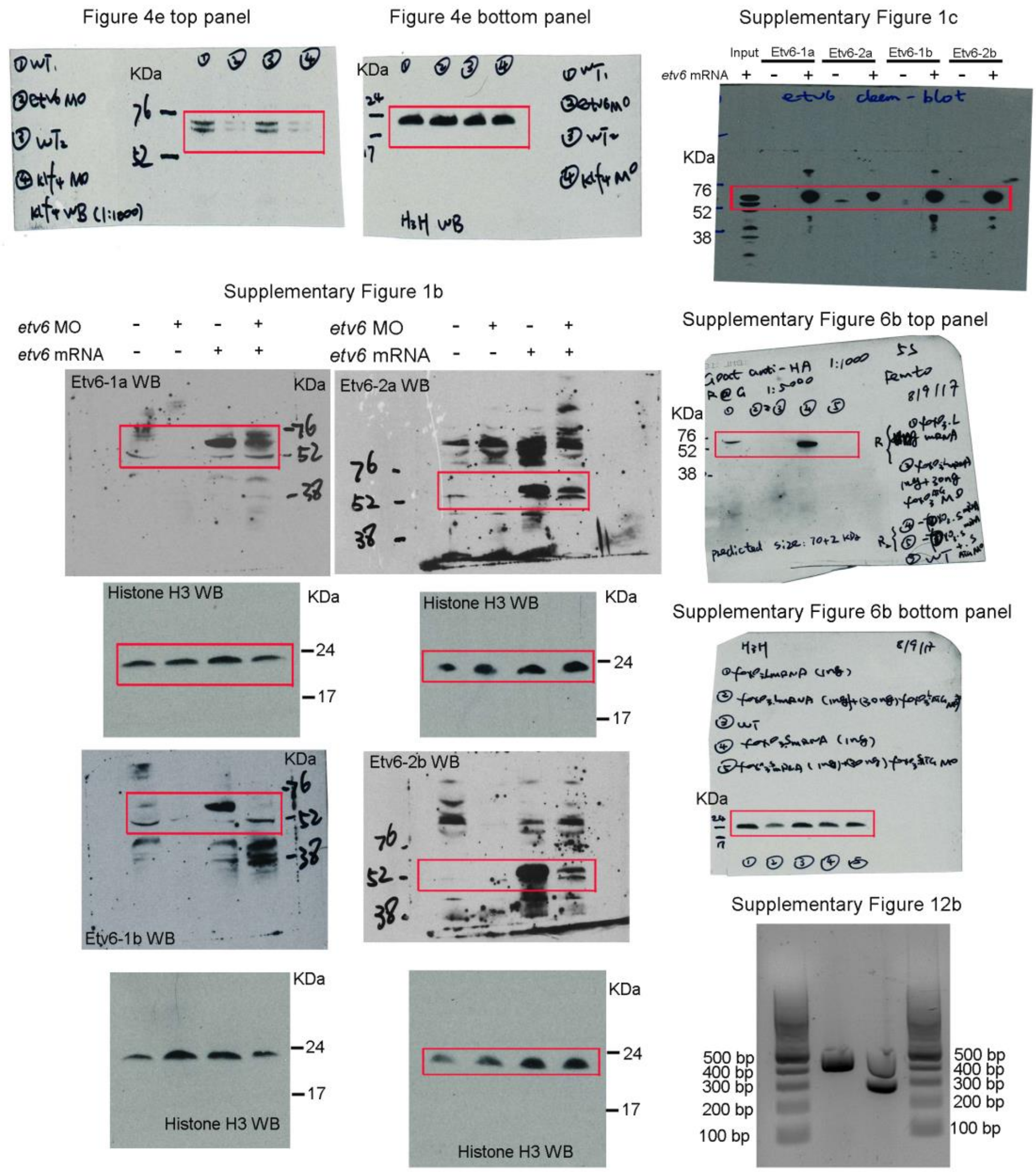

Supplementary Figure 14. Uncropped scans of western blots and agarose gels presented in Figure 4 and Supplementary Figures 1, 5 and 11.

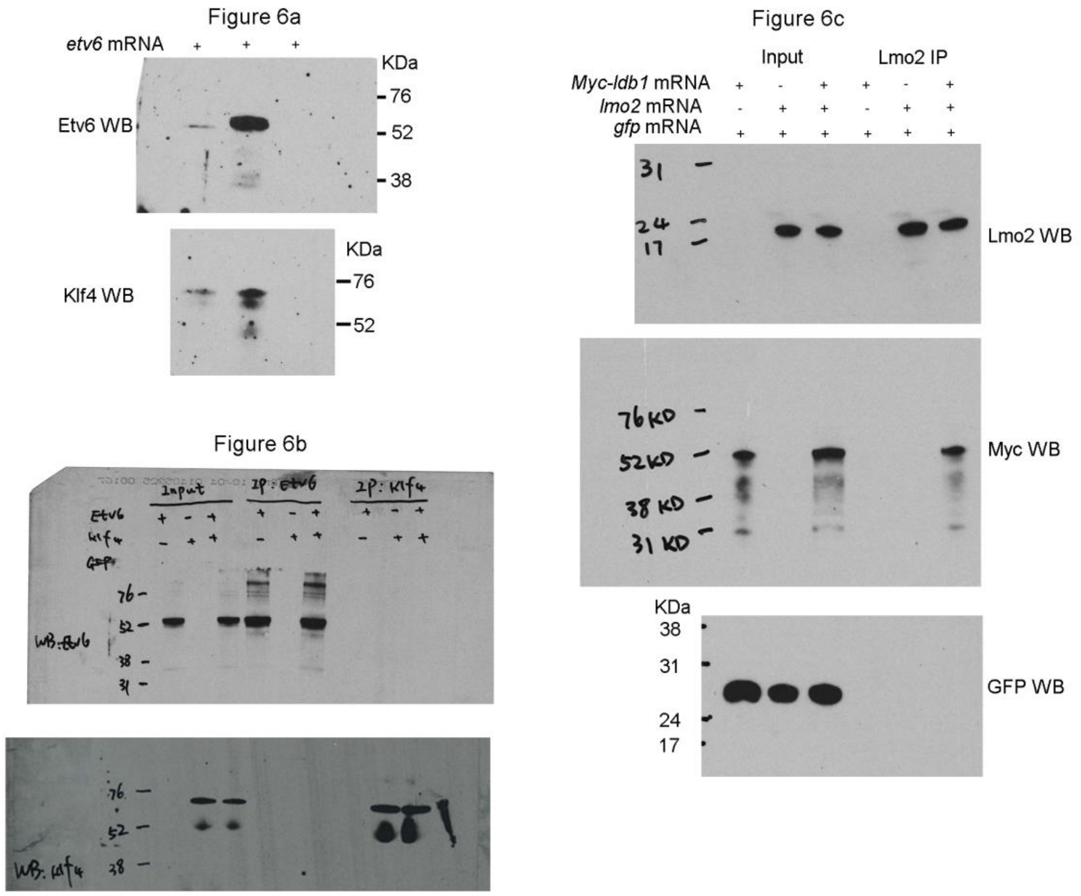

Supplementary Figure 15. Uncropped scans of western blots presented in Figure 6.

Supplementary Table 1. *De novo* motif analysis on the Etv6 ChIP-seq peaks located in the TSS region.

Rank	Motif	P-value	% of Targets	% of Background	Best Match
1	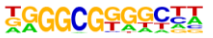	1e-922	66.01%	10.76%	Klf/Sp/Homer(0.90)
2	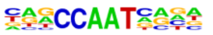	1e-334	30.22%	5.28%	NFY(CCAAT)/Promoter/Homer(0.94)
3	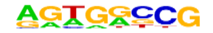	1e-106	21.32%	7.31%	MA0443.1_btd/Jaspar(0.690)
4	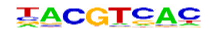	1e-80	17.26%	6.11%	bZIP_CREB /Jaspar(0.93)
5	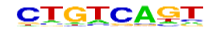	1e-74	62.36%	43.87%	MA0498.1_Meis1/Jaspar(0.907)
6	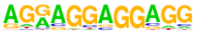	1e-62	15.29%	15.29%	MA0528.1_ZNF263/Jaspar(0.724)
7	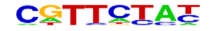	1e-47	31.16%	18.79%	MA0319.1_HSF1/Jaspar(0.762)
8	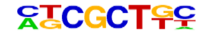	1e-45	15.87%	7.31%	POL010.1_DCE_S_III/Jaspar(0.761)
9	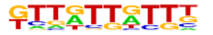	1e-39	17.79%	9.16%	PB0122.1_Foxk1_2/Jaspar(0.818)
10	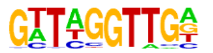	1e-37	3.53%	0.59%	AtMYB15(MYB)/Arabidopsis thaliana/AthaMap(0.676)
11	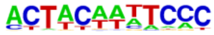	1e-32	3.49%	0.68%	GFY(?)/Promoter/Homer(0.928)
12	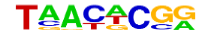	1e-31	6.93%	2.45%	STB5/STB5_YPD/Yeast(0.749)
13	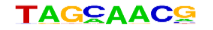	1e-31	10.74%	4.87%	PB0056.1_Rfxdc2_1/Jaspar(0.911)
14	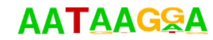	1e-28	8.94%	3.87%	MA0127.1_PEND/Jaspar(0.816)
15	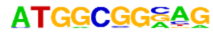	1e-27	5.78%	1.99%	YY1(Zf)/Promoter/Homer(0.760)
16	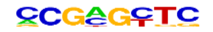	1e-26	11.28%	5.57%	MA0374.1_RSC3/Jaspar(0.698)
17	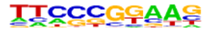	1e-18	2.34%	0.54%	Stat3(Stat) /Homer(0.898)
18	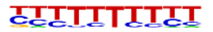	1e-17	3.90%	1.40%	SeqBias: polyA-repeat(0.888)
19	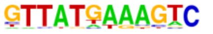	1e-15	1.15%	0.14%	nub /fly(0.692)
20	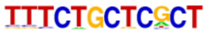	1e-14	0.62%	0.03%	MA0326.1_MAC1/Jaspar(0.659)
21	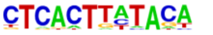	1e-9	0.37%	0.02%	MA0537.1_BLMP-1/Jaspar(0.607)
22	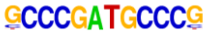	1e-5	0.16%	0.01%	MA0362.1_RDS2/Jaspar(0.645)

Supplementary Table 2. *De novo* motif analysis on Etv6 ChIP-seq peaks associated with the TSS region of genes repressed or activated by Etv6.

Motifs overrepresented in the Etv6 peaks associated with the TSS region of genes repressed by Etv6

Rank	Motif	P-value	% of Targets	% of Background	Best Match
1	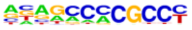	1e-65	68.97%	15.33%	Klf/Sp/Homer(0.87)
2	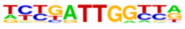	1e-24	21.67%	2.97%	NFY(CCAAT)/Promoter/Homer(0.93)
3	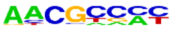	1e-12	16.75%	3.70%	hkb/fly(0.850)
4	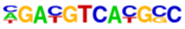	1e-12	2.46%	0.01%	CREB/Jaspar (0.74)
5	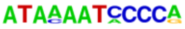	1e-12	2.46%	0.01%	Hox/Jaspar(0.68)
6	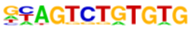	1e-12	4.43%	0.10%	Smad/Homer (0.62)

Motifs overrepresented in the Etv6 peaks associated with the TSS region of genes activated by Etv6

Rank	Motif	P-value	% of Targets	% of Background	Best Match
1	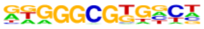	1e-123	66.37%	11.78%	Klf/Sp/Homer(0.89)
2	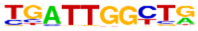	1e-46	23.98%	3.15%	NFY(CCAAT)/Promoter/Homer(0.91)
3	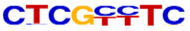	1e-26	23.68%	5.87%	MA0260.1_che-1/Jaspar(0.668)
4	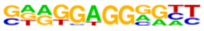	1e-23	19.59%	4.49%	Klf/Sp/Homer(0.804)
5	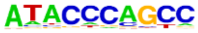	1e-21	12.57%	1.9%	PB0167.1_Sox13_2/Jaspar (0.67)
6	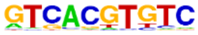	1e-17	19.30%	5.64%	NPAS2(bHLH) /Homer (0.84)
7	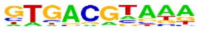	1e-16	8.77%	1.17%	bZIP_CREB /Jaspar (0.87)
8	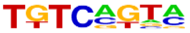	1e-16	20.18%	6.55%	MA0498.1_Meis/Jaspar (0.76)
9	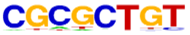	1e-14	16.37%	4.98%	MA0374.1_RSC3/Jaspar (0.72)
10	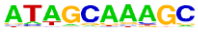	1e-13	17.84%	5.88%	Tcf3(HMG) /Homer (0.69)
11	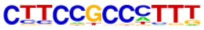	1e-13	6.14%	0.65%	ELF1(ETS) /Homer (0.65)
12	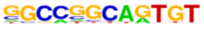	1e-12	3.80%	0.18%	ETS1(ETS)/Promoter/Homer (0.58)

Supplementary Table 3. Probes used for *in situ* hybridization. Probes were designed to target both L and S genes unless otherwise indicated.

Gene Name	Accession Number	Restriction Enzyme	RNA Polymerase	Reference
Meox2	L20432	SacII	SP6	This Report
Crim1	EU882845	ApaI	SP6	This Report
Sox18	BC072123	SalI or SmaI	T7	This Report
Vegfa.L		BamHI	T7	Cleaver et al., 1997[1]
Foxo3	AJ783964	SalI	T7	This Report
Klf4	BC055956	SalI or SacI	T7	This Report
Runx1		SalI	T7	Tracey et al., 1998[2]
Tal1		XhoI	SP6	Ciau-Uitz et al., 2010[3]
Etv6	EU760352	NotI or SacI	T7	Ciau-Uitz et al., 2010[3]

Supplementary Table 4. MO sequences.

Gene Name	MO sequence	References
Etv6	GTAACACTGGGCTGAAGACATTTCC	Ciau-Uitz et al., 2010[3]
Foxo3.L	TGCTTCTGCCATGCTGCGAGAAG	This report
Foxo3.S	GGCTTCTGCCATGGTGTGTGAAC	This report
Klf4	CTGCCTCATTAATCTGGGAGGGTCA	This report

Supplementary Table 5. Primer sequences for cloning.

Gene		Primer sequences
Etv6.S mRNA	Forward	actctagagatatcgagctcgaggtttctgtttcccagagg
	Reverse	acatcgtatggatatcgggatccgcattcatcctcctgatac
Foxo3.L mRNA	Forward	cgactctagacttctcgcagcatggcagaagcactgcc
	Reverse	agacgtcgacgcctggcacccagctc
Foxo3.S mRNA	Forward	cgactctagagttcacacaccatggcagaagccgtgcc
	Reverse	agacgtcgacgcctggcacccagctc
Klf4.L mRNA	Forward	cgactctagagccaccatgaggcagcagccaccc
	Reverse	agacgtcgacgaagtgtcttttcatgtgc
Klf4 talen test	Forward	tagtcactggctttggctgc
	Reverse	gcaggatactcacattgcct
Klf4 binding motif KO talen test	Forward	cgcacaccgaagcaattctc
	Reverse	ctcaatgcatcgcgcgtaaa
Klf4.L-luc	Forward	GCCGGTACCgagctggatgactgatagagg
	Reverse	ATCAAGATCTgaggctccttgtcagataga
Foxo3.L-luc	Forward	GCCGGTACCtttgctctgttcattgttttcc
	Reverse	ATCAAGATCTcgcgccagagggagccag
Foxo3.S-luc	Forward	GCCGGTACCaggccaacactgcattatattt
	Reverse	GCCAAGCTTacggggagggggttatgttta
Vegfa.L-luc	Forward	GCCGGTACCccacagccacactcccttcc
	Reverse	GCCAAGCTTaaagttacacacatatacacat
Vegfa.L-Foxo3 binding motif deletion-luc	Forward	ttttggaagagggacaaaagattt
	Reverse	tgtccctcttccaaaatgttggga

Supplementary Table 6. Primer sequences for RT-qPCR. Primers were designed to target both L and S genes.

Gene Name		Primer sequences
Foxo3	Forward	tccgctccgtgccctactt
	Reverse	cgtcttcttggtgcctttcc
Klf4	Forward	tgcggaaagacctataccaag
	Reverse	ttcggtagtggcgggtcag
Etv6	Forward	gccgcctactctgggattac
	Reverse	gcgcagagcccgtgacatc
Odc	Forward	ggcgacattgtgaaaaagca
	Reverse	cgggtttgcatagataatcc

Supplementary Table 7. Primer sequences for ChIP-qPCR. Primers were designed to target both L and S genes unless otherwise indicated.

Gene Name		Primer sequences
Foxo3 peak	Forward	agcctgtcaccctgcgcgcc
	Reverse	ccagggctcctccccttc
Foxo3 exon2	Forward	tgattctcccctgtcccctatg
	Reverse	tgctgtgaagaagtgagagaaatg
Klf4 peak	Forward	gcggggcttgtctccacgtgat
	Reverse	gccagggcaccggaac
Klf4 intron2	Forward	agcatctccagacagccaaagt
	Reverse	gtaggtggtgcaatatctcttaa
Vegfa.L peak	Forward	tgcagcctcagtgtcacataac
	Reverse	cccgggtctctcttcttacact
Vegfa.L intron1	Forward	ggccgacagttaagcgattca
	Reverse	cccccaccccaggaaatgat
Vegfa.L Promoter 2	Forward	ccacagccacactcccttccta
	Reverse	aaaaaattgctgcgctacatg

Supplementary Table 8. Plasmids used for *in vitro* mRNA generation.

Plasmid Name	Restriction Enzyme	RNA Polymerase	Reference
Etv6.S-HA	Sfi1	T3	This Report
Foxo3.L-HA	Sfi1	T3	This Report
Foxo3.S-HA	Sfi1	T3	This Report
Klf4.L-HA	Sfi1	T3	This Report
Ldb1-MYC	Not1	SP6	This Report
Lmo2	EcoRI	T3	Gering et al., 2003[4]
GFP-NLS	Not1	SP6	This Report

Supplementary references

1 Cleaver, O., Tonissen, K. F., Saha, M. S. & Krieg, P. A. Neovascularization of the Xenopus embryo. *Dev Dyn* **210**, 66-77 (1997).

2 Tracey WD Jr, Pepling ME, Horb ME, Thomsen GH & JP., G. A Xenopus homologue of AML1 reveals unexpected patterning mechanisms leading to the formation of embryonic blood. *Development* **125**, 1372-1380 (1998).

3 Ciau-Uitz, A., Pinheiro, P., Gupta, R., Enver, T. & Patient, R. Tel1/ETV6 Specifies Blood Stem Cells through the Agency of VEGF Signaling. *Developmental Cell* **18**, 569-578 (2010).

4 Gering, M., Yamada, Y., Rabbitts, T. H. & Patient, R. K. Lmo2 and Scl/Tal1 convert non-axial mesoderm into haemangioblasts which differentiate into endothelial cells in the absence of Gata1. *Development* **130**, 6187-6199 (2003).
