# Supplementary material for: Etv6 activates vegfa expression through positive and negative transcriptional regulatory networks in Xenopus embryos
Source: Nat Commun. 2019 Mar 6;10:1083. doi: 10.1038/s41467-019-09050-y (PMC6403364; doi:10.1038/s41467-019-09050-y)
Supplement: Supplementary file 9 — Reporting Summary [file 41467_2019_9050_MOESM9_ESM.pdf]

nature research

Corresponding author(s): Roger Patient, Aldo Ciau-Uitz & Catherine Porcher

Reporting Summary

Nature Research wishes to improve the reproducibility of the work that we publish. This form provides structure for consistency and transparency in reporting. For further information on Nature Research policies, see Authors & Referees and the Editorial Policy Checklist.

Statistical parameters

When statistical analyses are reported, confirm that the following items are present in the relevant location (e.g. figure legend, table legend, main text, or Methods section).

n/a	Confirmed	
☐	☒	The exact sample size (*n*) for each experimental group/condition, given as a discrete number and unit of measurement
☐	☒	An indication of whether measurements were taken from distinct samples or whether the same sample was measured repeatedly
☐	☒	The statistical test(s) used AND whether they are one- or two-sided<br>*Only common tests should be described solely by name; describe more complex techniques in the Methods section.*
☒	☐	A description of all covariates tested
☒	☐	A description of any assumptions or corrections, such as tests of normality and adjustment for multiple comparisons
☐	☒	A full description of the statistics including central tendency (e.g. means) or other basic estimates (e.g. regression coefficient) AND variation (e.g. standard deviation) or associated estimates of uncertainty (e.g. confidence intervals)
☐	☒	For null hypothesis testing, the test statistic (e.g. *F*, *t*, *r*) with confidence intervals, effect sizes, degrees of freedom and *P* value noted<br>*Give P values as exact values whenever suitable.*
☒	☐	For Bayesian analysis, information on the choice of priors and Markov chain Monte Carlo settings
☒	☐	For hierarchical and complex designs, identification of the appropriate level for tests and full reporting of outcomes
☒	☐	Estimates of effect sizes (e.g. Cohen's *d*, Pearson's *r*), indicating how they were calculated
☐	☒	Clearly defined error bars<br>*State explicitly what error bars represent (e.g. SD, SE, CI)*

Our web collection on statistics for biologists may be useful.

Software and code

Policy information about availability of computer code

Data collection	ACT-1 for DXM1200 (Nikon)
Data analysis	Adobe Photoshop CS4, Adobe Illustrator CS4, GraphPad Prism 8. Details of NGS data analysis are in the GEO submission and "Methods" section in the manuscript

For manuscripts utilizing custom algorithms or software that are central to the research but not yet described in published literature, software must be made available to editors/reviewers upon request. We strongly encourage code deposition in a community repository (e.g. GitHub). See the Nature Research guidelines for submitting code & software for further information.

Data

Policy information about availability of data

All manuscripts must include a data availability statement. This statement should provide the following information, where applicable:
- Accession codes, unique identifiers, or web links for publicly available datasets
- A list of figures that have associated raw data
- A description of any restrictions on data availability

All ChIP-seq and RNA-seq datasets have been deposited online in the Gene Expression Omnibus (GEO) under accession number GSE115225. All other data supporting the results of this study are available within the article and its supplementary information files and from the corresponding authors upon request.

Field-specific reporting

Please select the best fit for your research. If you are not sure, read the appropriate sections before making your selection.

☒ Life sciences ☐ Behavioural & social sciences ☐ Ecological, evolutionary & environmental sciences

For a reference copy of the document with all sections, see nature.com/authors/policies/ReportingSummary-flat.pdf

Life sciences study design

All studies must disclose on these points even when the disclosure is negative.

Sample size	No statistical methods were used to predetermine sample size. Sample sizes were predetermined on the basis of published studies. Number of embryos for WISH were maximum with evenly staining.
Data exclusions	No data were excluded in this study.
Replication	All experiments in this study were reproduced at least three times using three biological replicates with the same protocol.
Randomization	Xenopus laevis embryos were allocated to experiment groups on the basis of different treatments and randomized within the given group.
Blinding	The investigators were not blinded to the group allocation during experiments and outcome assessment.

Behavioural & social sciences study design

All studies must disclose on these points even when the disclosure is negative.

Study description	*Briefly describe the study type including whether data are quantitative, qualitative, or mixed-methods (e.g. qualitative cross-sectional, quantitative experimental, mixed-methods case study).*
Research sample	*State the research sample (e.g. Harvard university undergraduates, villagers in rural India) and provide relevant demographic information (e.g. age, sex) and indicate whether the sample is representative. Provide a rationale for the study sample chosen. For studies involving existing datasets, please describe the dataset and source.*
Sampling strategy	*Describe the sampling procedure (e.g. random, snowball, stratified, convenience). Describe the statistical methods that were used to predetermine sample size OR if no sample-size calculation was performed, describe how sample sizes were chosen and provide a rationale for why these sample sizes are sufficient. For qualitative data, please indicate whether data saturation was considered, and what criteria were used to decide that no further sampling was needed.*
Data collection	*Provide details about the data collection procedure, including the instruments or devices used to record the data (e.g. pen and paper, computer, eye tracker, video or audio equipment) whether anyone was present besides the participant(s) and the researcher, and whether the researcher was blind to experimental condition and/or the study hypothesis during data collection.*
Timing	*Indicate the start and stop dates of data collection. If there is a gap between collection periods, state the dates for each sample cohort.*
Data exclusions	*If no data were excluded from the analyses, state so OR if data were excluded, provide the exact number of exclusions and the rationale behind them, indicating whether exclusion criteria were pre-established.*
Non-participation	*State how many participants dropped out/declined participation and the reason(s) given OR provide response rate OR state that no participants dropped out/declined participation.*
Randomization	*If participants were not allocated into experimental groups, state so OR describe how participants were allocated to groups, and if allocation was not random, describe how covariates were controlled.*

Ecological, evolutionary & environmental sciences study design

All studies must disclose on these points even when the disclosure is negative.

Study description	*Briefly describe the study. For quantitative data include treatment factors and interactions, design structure (e.g. factorial, nested, hierarchical), nature and number of experimental units and replicates.*
Research sample	*Describe the research sample (e.g. a group of tagged Passer domesticus, all Stenocereus thurberi within Organ Pipe Cactus National Monument), and provide a rationale for the sample choice. When relevant, describe the organism taxa, source, sex, age range and any manipulations. State what population the sample is meant to represent when applicable. For studies involving existing datasets, describe the data and its source.*

Sampling strategy | *Note the sampling procedure. Describe the statistical methods that were used to predetermine sample size OR if no sample-size calculation was performed, describe how sample sizes were chosen and provide a rationale for why these sample sizes are sufficient.*

Data collection | *Describe the data collection procedure, including who recorded the data and how.*

Timing and spatial scale | *Indicate the start and stop dates of data collection, noting the frequency and periodicity of sampling and providing a rationale for these choices. If there is a gap between collection periods, state the dates for each sample cohort. Specify the spatial scale from which the data are taken*

Data exclusions | *If no data were excluded from the analyses, state so OR if data were excluded, describe the exclusions and the rationale behind them, indicating whether exclusion criteria were pre-established.*

Reproducibility | *Describe the measures taken to verify the reproducibility of experimental findings. For each experiment, note whether any attempts to repeat the experiment failed OR state that all attempts to repeat the experiment were successful.*

Randomization | *Describe how samples/organisms/participants were allocated into groups. If allocation was not random, describe how covariates were controlled. If this is not relevant to your study, explain why.*

Blinding | *Describe the extent of blinding used during data acquisition and analysis. If blinding was not possible, describe why OR explain why blinding was not relevant to your study.*

Did the study involve field work? [ ] Yes [ ] No

Field work, collection and transport

Field conditions | *Describe the study conditions for field work, providing relevant parameters (e.g. temperature, rainfall).*

Location | *State the location of the sampling or experiment, providing relevant parameters (e.g. latitude and longitude, elevation, water depth).*

Access and import/export | *Describe the efforts you have made to access habitats and to collect and import/export your samples in a responsible manner and in compliance with local, national and international laws, noting any permits that were obtained (give the name of the issuing authority, the date of issue, and any identifying information).*

Disturbance | *Describe any disturbance caused by the study and how it was minimized.*

Reporting for specific materials, systems and methods

Materials & experimental systems

n/a	Involved in the study
[x]	[ ] Unique biological materials
[ ]	[x] Antibodies
[x]	[ ] Eukaryotic cell lines
[x]	[ ] Palaeontology
[ ]	[x] Animals and other organisms
[x]	[ ] Human research participants

Methods

n/a	Involved in the study
[ ]	[x] ChIP-seq
[x]	[ ] Flow cytometry
[x]	[ ] MRI-based neuroimaging

Unique biological materials

Policy information about availability of materials

Obtaining unique materials | *Describe any restrictions on the availability of unique materials OR confirm that all unique materials used are readily available from the authors or from standard commercial sources (and specify these sources).*

Antibodies

Antibodies used | Klf4 antibody (Abcam, ab106629)
Foxo3 antibody (Thermo Fisher Scientific, Cat#720128)
HA antibody (Santa Cruz Biotechnology, HA-probe (Y-11)-G)
Histone H3 antibody (Abcam, ab1791)
Myc antibody (Santa Cruz Biotechnology, sc-40)
GFP antibody (Millipore, MAB3580)

	Lmo2 antibody (Abcam, ab72841)<br>Etv6 antibodies (NovoPro Bioscience Inc., custom made)
Validation	Etv6 antibodies were validated using western blot and co-immunoprecipitation experiments. Klf4, Foxo3, HA, Histone H3, Myc, GFP and Lmo2 antibodies were validated for use in the study. See details in "Methods" section in the manuscript.

Eukaryotic cell lines

Policy information about cell lines

Cell line source(s)	*State the source of each cell line used.*
Authentication	*Describe the authentication procedures for each cell line used OR declare that none of the cell lines used were authenticated.*
Mycoplasma contamination	*Confirm that all cell lines tested negative for mycoplasma contamination OR describe the results of the testing for mycoplasma contamination OR declare that the cell lines were not tested for mycoplasma contamination.*
Commonly misidentified lines<br>(See ICLAC register)	*Name any commonly misidentified cell lines used in the study and provide a rationale for their use.*

Palaeontology

Specimen provenance	*Provide provenance information for specimens and describe permits that were obtained for the work (including the name of the issuing authority, the date of issue, and any identifying information).*
Specimen deposition	*Indicate where the specimens have been deposited to permit free access by other researchers.*
Dating methods	*If new dates are provided, describe how they were obtained (e.g. collection, storage, sample pretreatment and measurement), where they were obtained (i.e. lab name), the calibration program and the protocol for quality assurance OR state that no new dates are provided.*

☐ Tick this box to confirm that the raw and calibrated dates are available in the paper or in Supplementary Information.

Animals and other organisms

Policy information about studies involving animals; ARRIVE guidelines recommended for reporting animal research

Laboratory animals	J-strain Xenopus laevis embryos were obtained, cultured and injected as previously described (Walmsley et al., 2005). Animal experiments were performed according to UK Home Office regulations under the appropriate project licence and approval of the University of Oxford Animal Welfare and Ethical Review Body.
Wild animals	The study did not involve wild animals
Field-collected samples	The study did not involve samples collected from the field.

Human research participants

Policy information about studies involving human research participants

Population characteristics	*Describe the covariate-relevant population characteristics of the human research participants (e.g. age, gender, genotypic information, past and current diagnosis and treatment categories). If you filled out the behavioural & social sciences study design questions and have nothing to add here, write "See above."*
Recruitment	*Describe how participants were recruited. Outline any potential self-selection bias or other biases that may be present and how these are likely to impact results.*

ChIP-seq

Data deposition

☒ Confirm that both raw and final processed data have been deposited in a public database such as GEO.

☒ Confirm that you have deposited or provided access to graph files (e.g. BED files) for the called peaks.

Data access links<br>*May remain private before publication.*	https://www.ncbi.nlm.nih.gov/geo/query/acc.cgi?acc=GSE115225
Files in database submission	etv6-chip1_S10_L001_R1_001.fastq.gz<br>etv6-chip1_S10_L002_R1_001.fastq.gz<br>etv6-chip1_S10_L003_R1_001.fastq.gz

etv6-chip1_S10_L004_R1_001.fastq.gz
etv6-chip1_S10_L001_R2_001.fastq.gz
etv6-chip1_S10_L002_R2_001.fastq.gz
etv6-chip1_S10_L003_R2_001.fastq.gz
etv6-chip1_S10_L004_R2_001.fastq.gz
etv6-chip2_S2_L001_R1_001.fastq.gz
etv6-chip2_S2_L002_R1_001.fastq.gz
etv6-chip2_S2_L003_R1_001.fastq.gz
etv6-chip2_S2_L004_R1_001.fastq.gz
etv6-chip2_S2_L001_R2_001.fastq.gz
etv6-chip2_S2_L002_R2_001.fastq.gz
etv6-chip2_S2_L003_R2_001.fastq.gz
etv6-chip2_S2_L004_R2_001.fastq.gz
etv6-chip3_S4_L001_R1_001.fastq.gz
etv6-chip3_S4_L002_R1_001.fastq.gz
etv6-chip3_S4_L003_R1_001.fastq.gz
etv6-chip3_S4_L004_R1_001.fastq.gz
etv6-chip3_S4_L001_R2_001.fastq.gz
etv6-chip3_S4_L002_R2_001.fastq.gz
etv6-chip3_S4_L003_R2_001.fastq.gz
etv6-chip3_S4_L004_R2_001.fastq.gz
etv6-Input1_S3_L001_R1_001.fastq.gz
etv6-Input1_S3_L002_R1_001.fastq.gz
etv6-Input1_S3_L003_R1_001.fastq.gz
etv6-Input1_S3_L004_R1_001.fastq.gz
etv6-Input1_S3_L001_R2_001.fastq.gz
etv6-Input1_S3_L002_R2_001.fastq.gz
etv6-Input1_S3_L003_R2_001.fastq.gz
etv6-Input1_S3_L004_R2_001.fastq.gz
etv6-Input2_S11_L001_R1_001.fastq.gz
etv6-Input2_S11_L002_R1_001.fastq.gz
etv6-Input2_S11_L003_R1_001.fastq.gz
etv6-Input2_S11_L004_R1_001.fastq.gz
etv6-Input2_S11_L001_R2_001.fastq.gz
etv6-Input2_S11_L002_R2_001.fastq.gz
etv6-Input2_S11_L003_R2_001.fastq.gz
etv6-Input2_S11_L004_R2_001.fastq.gz
etv6-Input3_S9_L001_R1_001.fastq.gz
etv6-Input3_S9_L002_R1_001.fastq.gz
etv6-Input3_S9_L003_R1_001.fastq.gz
etv6-Input3_S9_L004_R1_001.fastq.gz
etv6-Input3_S9_L001_R2_001.fastq.gz
etv6-Input3_S9_L002_R2_001.fastq.gz
etv6-Input3_S9_L003_R2_001.fastq.gz
etv6-Input3_S9_L004_R2_001.fastq.gz
consensus_peacks.bed.gz

Genome browser session
(e.g. UCSC)

Provide a link to an anonymized genome browser session for "Initial submission" and "Revised version" documents only, to enable peer review. Write "no longer applicable" for "Final submission" documents.

Methodology

Replicates

There are three biological replicates for Etv6 ChIP-seq samples (Etv6 ChIP1/ChIP2/ChIP3) with corresponding inputs as control (input1/input2/input3).

Sequencing depth

Sample, Read Depth, Uniquely Mapped, Read Length, Single/Paired-End
Input1 30,718,918 22,809,969 40 paried-end
Input2 34,714,248 25,949,542 40 paired-end
input3 43,900,158 32,849,215 40 paired-end
Etv6 ChIP1 65,447,426 35,513,440 40 paired-end
Etv6 ChIP2 75,500,184 40,612,772 40 paired-end
Etv6 ChIP3 51,827,454 20,737,303 40 paired-end

Antibodies

Etv6 antibody (NovoPro Bioscience Inc., custom made). See details in Methods section and Supplementary Figure 1.

Peak calling parameters

Peaks were analyzed using MACS2 peak calling software at default thresholds, with the input samples as control for each replicate.

Data quality

Raw sequence reads were checked for base quality, trimmed and filtered to exclude adapters using Trimmomatic (Version 0.32), and then mapped to the X. laevis V9.1 with BWA version 0.7.12.

Software

Raw sequence reads were trimmed using Trimmomatic (Version 0.32), and mapped to the X. laevis V9.1 with BWA version 0.7.12. Peaks were analyzed using MACS2 peak calling software at default thresholds, with the input samples as control for

each replicate. Peaks consistent between replicates were identified using DiffBind (Bioconductor package), those located in TSS regions were identified using Homer software and used to perform TFs binding site analysis with Homer software (findMotifsGenome.pl). For the TFs binding site prediction, DNA sequences under the Etv6 peak were analyzed using Jaspar. ChIP-seq data was visualized on the Integrative Genome Viewer (IGV).

Flow Cytometry

Plots

Confirm that:

- [ ] The axis labels state the marker and fluorochrome used (e.g. CD4-FITC).
- [ ] The axis scales are clearly visible. Include numbers along axes only for bottom left plot of group (a 'group' is an analysis of identical markers).
- [ ] All plots are contour plots with outliers or pseudocolor plots.
- [ ] A numerical value for number of cells or percentage (with statistics) is provided.

Methodology

Sample preparation	*Describe the sample preparation, detailing the biological source of the cells and any tissue processing steps used.*
Instrument	*Identify the instrument used for data collection, specifying make and model number.*
Software	*Describe the software used to collect and analyze the flow cytometry data. For custom code that has been deposited into a community repository, provide accession details.*
Cell population abundance	*Describe the abundance of the relevant cell populations within post-sort fractions, providing details on the purity of the samples and how it was determined.*
Gating strategy	*Describe the gating strategy used for all relevant experiments, specifying the preliminary FSC/SSC gates of the starting cell population, indicating where boundaries between "positive" and "negative" staining cell populations are defined.*

- [ ] Tick this box to confirm that a figure exemplifying the gating strategy is provided in the Supplementary Information.

Magnetic resonance imaging

Experimental design

Design type	*Indicate task or resting state; event-related or block design.*
Design specifications	*Specify the number of blocks, trials or experimental units per session and/or subject, and specify the length of each trial or block (if trials are blocked) and interval between trials.*
Behavioral performance measures	*State number and/or type of variables recorded (e.g. correct button press, response time) and what statistics were used to establish that the subjects were performing the task as expected (e.g. mean, range, and/or standard deviation across subjects).*

Acquisition

Imaging type(s)	*Specify: functional, structural, diffusion, perfusion.*
Field strength	*Specify in Tesla*
Sequence & imaging parameters	*Specify the pulse sequence type (gradient echo, spin echo, etc.), imaging type (EPI, spiral, etc.), field of view, matrix size, slice thickness, orientation and TE/TR/flip angle.*
Area of acquisition	*State whether a whole brain scan was used OR define the area of acquisition, describing how the region was determined.*

Diffusion MRI ☐ Used ☐ Not used

Preprocessing

Preprocessing software	*Provide detail on software version and revision number and on specific parameters (model/functions, brain extraction, segmentation, smoothing kernel size, etc.).*
Normalization	*If data were normalized/standardized, describe the approach(es): specify linear or non-linear and define image types used for transformation OR indicate that data were not normalized and explain rationale for lack of normalization.*
Normalization template	*Describe the template used for normalization/transformation, specifying subject space or group standardized space (e.g. original Talairach, MNI305, ICBM152) OR indicate that the data were not normalized.*
Noise and artifact removal	*Describe your procedure(s) for artifact and structured noise removal, specifying motion parameters, tissue signals and*

Noise and artifact removal | *physiological signals (heart rate, respiration).*

Volume censoring | *Define your software and/or method and criteria for volume censoring, and state the extent of such censoring.*

Statistical modeling & inference

Model type and settings | *Specify type (mass univariate, multivariate, RSA, predictive, etc.) and describe essential details of the model at the first and second levels (e.g. fixed, random or mixed effects; drift or auto-correlation).*

Effect(s) tested | *Define precise effect in terms of the task or stimulus conditions instead of psychological concepts and indicate whether ANOVA or factorial designs were used.*

Specify type of analysis: ☐ Whole brain ☐ ROI-based ☐ Both

Statistic type for inference (See Eklund et al. 2016) | *Specify voxel-wise or cluster-wise and report all relevant parameters for cluster-wise methods.*

Correction | *Describe the type of correction and how it is obtained for multiple comparisons (e.g. FWE, FDR, permutation or Monte Carlo).*

Models & analysis

n/a	Involved in the study
☐	☐ Functional and/or effective connectivity
☐	☐ Graph analysis
☐	☐ Multivariate modeling or predictive analysis

Functional and/or effective connectivity | *Report the measures of dependence used and the model details (e.g. Pearson correlation, partial correlation, mutual information).*

Graph analysis | *Report the dependent variable and connectivity measure, specifying weighted graph or binarized graph, subject- or group-level, and the global and/or node summaries used (e.g. clustering coefficient, efficiency, etc.).*

Multivariate modeling and predictive analysis | *Specify independent variables, features extraction and dimension reduction, model, training and evaluation metrics.*
