# Supplementary material for: Etv6 activates vegfa expression through positive and negative transcriptional regulatory networks in Xenopus embryos
Source: Nat Commun. 2019 Mar 6;10:1083. doi: 10.1038/s41467-019-09050-y (PMC6403364; doi:10.1038/s41467-019-09050-y)
Supplement: Supplementary file 10 — Description of Additional Supplementary Files [file 41467_2019_9050_MOESM10_ESM.docx]

**Title: Supplementary Data : Etv6 ChIP-seq consistent peaks**

**Description:** Chromosomal coordinates of the 9,128 Etv6 peaks common across the 3 ChIP-seq replicates.

**Title: Supplementary Data 2: *De novo* motif analysis on all Etv6 ChIP-seq peaks**

**Description:** Consensus DNA motifs significantly enriched under the 9,128 Etv6 peaks using HOMER software.

**Title: Supplementary Data 3: Etv6-regulated transcriptome in the somites**

**Description:** 5,186 differentially expressed genes (FDR<0.05) were identified by RNA-seq between WT and Etv6-deficient somites.

**Title: Supplementary Data 4: Gene Ontology analysis of Etv6-regulated transcriptome**

**Description:** Gene Ontology analysis was performed on the 5,186 genes differentially expressed between WT and Etv6-deficient somites using DAVID Bioinformatics Resources.

**Title: Supplementary Data 5: Etv6 putative direct targets**

**Description:** 540 genes are bound by Etv6 at their TSS and differentially expressed between WT and Etv6-deficient somites. The table lists their transcript Id, name, log fold-change of expression and the chromosomal coordinate of the Etv6 peaks at their TSS.

**Title: Supplementary Data 6**: **Predicted TF binding sites in *vegfa* promoter region using Jaspar database.**

**Description:** The sequence of the *vegfa* promoter region was submitted to the Jaspar database to identify putative TF binding sites.
